# Mechanically Ventilated Patients Shed High-Titer Live Severe Acute Respiratory Syndrome Coronavirus 2 (SARS-CoV-2) for Extended Periods From Both the Upper and Lower Respiratory Tract

**DOI:** 10.1093/cid/ciac170

**Published:** 2022-03-01

**Authors:** Zack Saud, Mark Ponsford, Kirsten Bentley, Jade M Cole, Manish Pandey, Stephen Jolles, Chris Fegan, Ian Humphreys, Matt P Wise, Richard Stanton

**Affiliations:** Division of Infection and Immunity, School of Medicine, Cardiff University, Cardiff, United Kingdom; Division of Infection and Immunity, School of Medicine, Cardiff University, Cardiff, United Kingdom; Immunodeficiency Centre for Wales, University Hospital of Wales, Cardiff, United Kingdom; Division of Infection and Immunity, School of Medicine, Cardiff University, Cardiff, United Kingdom; Adult Critical Care, University Hospital of Wales, Heath Park, Cardiff, United Kingdomand; Adult Critical Care, University Hospital of Wales, Heath Park, Cardiff, United Kingdomand; Immunodeficiency Centre for Wales, University Hospital of Wales, Cardiff, United Kingdom; Department of Hematology, University Hospital of Wales, Cardiff, United Kingdom; Division of Infection and Immunity, School of Medicine, Cardiff University, Cardiff, United Kingdom; Adult Critical Care, University Hospital of Wales, Heath Park, Cardiff, United Kingdomand; Division of Infection and Immunity, School of Medicine, Cardiff University, Cardiff, United Kingdom

**Keywords:** SARS-CoV-2, plaque assay, qPCR, intensive care unit, viral load

## Abstract

**Background:**

SARS-CoV-2 infection can lead to severe acute respiratory distress syndrome needing intensive care admission and may lead to death. As a virus that transmits by respiratory droplets and aerosols, determining the duration of viable virus shedding from the respiratory tract is critical for patient prognosis, and informs infection-control measures both within healthcare settings and the public domain.

**Methods:**

We prospectively examined upper and lower airway respiratory secretions for both viral RNA and infectious virions in mechanically ventilated patients admitted to the intensive care unit (ICU) of the University Hospital of Wales. Samples were taken from the oral cavity (saliva), oropharynx (subglottic aspirate), or lower respiratory tract (nondirected bronchoalveolar lavage [NBAL] or bronchoalveolar lavage [BAL]) and analyzed by both quantitative PCR (qPCR) and plaque assay.

**Results:**

117 samples were obtained from 25 patients. qPCR showed extremely high rates of positivity across all sample types; however, live virus was far more common in saliva (68%) than in BAL/NBAL (32%). Average titers of live virus were higher in subglottic aspirates (4.5 × 10^7^) than in saliva (2.2 × 10^6^) or BAL/NBAL (8.5 × 10^6^) and reached >10^8^ PFU/mL in some samples. The longest duration of shedding was 98 days, while most patients (14/25) shed live virus for ≥20 days.

**Conclusions:**

ICU patients infected with SARS-CoV-2 can shed high titers of virus both in the upper and lower respiratory tract and tend to be prolonged shedders. This information is important for decision making around cohorting patients, de-escalation of personal protective equipment, and undertaking potential aerosol-generating procedures.

## Teaser

Patients on intensive therapy infected with SARS-CoV-2 tend to be prolonged shedders, excreting virus for far beyond the time periods specified in current guidelines, and live virus titers can be extremely high in both the upper and lower respiratory tracts.

The coronavirus disease 2019 (COVID-19) pandemic has resulted in a global human death toll of 5.36 million (as of 21 December 2021) [1]. Early symptoms include a dry cough, exertional shortness of breath, fatigue, lethargy, diarrhea, and high-grade fever [2]; and in 10–15% of cases, this can progress to severe pneumonia needing hospitalization. In 1–2% of cases the disease can lead to severe acute respiratory distress syndrome (ARDS) needing intensive care unit (ICU) admission and may lead to death [3]. As a virus that transmits by respiratory droplets and aerosols, determining the duration of viable virus shedding from the respiratory tract is critical for patient prognosis, and informs infection-control measures both within healthcare settings and the public domain [4]. Although symptoms may persist for weeks or even months post-infection, shedding of infectious viral particles almost never occurs beyond 10 days of symptom onset, even in hospitalized patients [5]. In a meta-analysis including more than 5000 SARS-CoV-2–infected individuals, viral RNA was detectable up to 83 days in the upper respiratory tract, but no study detected live virus beyond day 9 of illness [5]. However, this analysis was performed prior to the introduction of immunosuppressive agents as standard of care for individuals hospitalized with severe respiratory complications of COVID-19 [6]. Given emerging evidence that infectious virions can be recovered from individuals with acquired and inherited forms of immunodeficiency months after symptom onset [7–10], we investigated whether adults requiring admission to the ICU who are subject to both infection-mediated immune dysregulation [11] and iatrogenic immunosuppression [6] exhibited prolonged viral shedding. Furthermore, no study has investigated whether the 9-day “cutoff” for live virus isolation applies to the lower respiratory tract or airways.

We examined upper and lower airway respiratory secretions in mechanically ventilated patients with COVID-19 admitted to the ICU of the University Hospital of Wales for titers of infectious severe acute respiratory syndrome coronavirus 2 (SARS-CoV-2), and compared these to quantitative polymerase chain reaction (qPCR). We show that infectious viral particles are readily recoverable from saliva and that these patients can secrete extremely high levels of live SARS-CoV-2 from multiple sites in the respiratory tract well beyond the 20-day isolation period currently recommended by the Centers for Disease Control and Prevention (CDC) for patients with severe COVID-19 [12], and thus represent a nosocomial reservoir of infection.

## METHODS

### Sample Collection

Saliva was collected using Neutral Salivettes (Sarstedt, Germany), which were placed against the buccal mucosa for 2 minutes and then spun (2000 × *g*) to collect supernatant, or washed with Dulbecco’s modified Eagle medium (DMEM) if no supernatant was present. Subglottic endotracheal tubes are used in many ICUs as they reduce ventilator-associated pneumonia. Subglottic aspirates represent an accumulation of oropharyngeal secretions that accumulate above the endotracheal cuff. Bronchoalveolar lavage (BAL) was undertaken using a disposable Ambu aScope 4 and Broncho Sampler Set (Ambu UK) with lavage of up to 80 mL of sterile saline; alternatively, a nondirected BAL (NBAL) was performed by inserting a suction catheter into the lung until resistance was met and 20 mL of sterile saline inserted and slowly withdrawn. All patients received evidence-based treatment as per published health board or ICU directorate guidelines. Samples were transferred to the BSL3 laboratory and processed within 4 hours. Baseline characteristics and treatments are shown in Table 1.

**Table 1.** Baseline Characteristics of and Treatments for Patients

Variable	Values
Age, median (interquartile range), y	59 (50–68)
Female sex, n (%)	9 (36)
In-hospital mortality, n (%)	16 (64)
Received dexamethasone treatment, n (%)	23 (92)
Received remdesivir treatment, n (%)	15 (60)
Received tocilizumab treatment, n (%)	10 (40)

### Trial Design

Sample collection (20 October 2020–8 August 2020) was undertaken as a service evaluation to see if virus could be measured in respiratory tract samples as an alternative to reverse transcription–qPCR (RT-qPCR). From 1 February 2021–31 March 2021, participants were enrolled in ENLIST (REC reference 20/YH/0309) and consent taken from a relative or legal representative. Sampling was prospective, and weekly unless clinically indicated otherwise. Inclusion criteria were as follows: (1) age 18 years or older, (2) clinical presentation of COVID-19, (3) PCR positive for SARS-CoV-2, and (4) admitted to the ICU requiring invasive mechanical ventilation. The exclusion criterion was age younger than 18 years.

### Plaque Assays

Cells were grown in DMEM containing 10% (vol/vol) fetal calf serum (FCS) and incubated at 37°C in 5% CO_2_. Plaque assays utilized VeroE6 expressing angiotensin converting enzyme 2 (ACE2) and transmembrane protease serine 2 (TMPRSS2) to enhance virus entry [13]. Serial dilutions of sample were applied to cells for 1 hour at 37°C with rocking. Cells were overlaid with DMEM containing 2% FCS, 1.2% Avicel (DuPont, USA), 50 μg/mL Gentamycin (Fisher Scientific, UK), and 2.5 μg/mL Amphotericin-B (Sigma Aldrich, UK). After 72 hours, the overlay was removed, the monolayer washed, fixed with 100% methanol, stained with 25% (vol/vol) methanol and 0.5% (wt/vol) Crystal Violet, then washed, and plaques enumerated.

### RNA Extraction

Samples (100 μL) were incubated with 10 μL of Proteinase K (Qiagen UK) for 15 minutes at room temperature, then incubated at 70°C for 15 minutes to inactivate enzyme. Ten microliters of RQ1 DNase buffer (Promega, UK) and 10 μL of RQ1 DNase (Promega, UK) were added, then incubated at 37°C for 30 minutes. RNA was extracted using the QIAmp Viral RNA Minikit (Qiagen, UK), and eluted in 60 μL of buffer AVE.

### Quantitative PCR

RT-qPCR for SARS-CoV-2 was carried out using E-gene targeting primers and probe: ACAGGTACGTTAATAGTTAATAGCGT, ATATTGCAGCAGTACGCACACA, FAM-ACACTAGCCATCCTTACTGCGCTTCG-BBQ. Copy number was quantified using a control plasmid (pEX-A128-nCoV_E_Sarbeco; Eurofins Genomics, Germany). RNA quality was assessed by RNAse P detection [14] using primers and probe: AGATTTGGACCTGCGAGCG, GAGCGGCTGTCTCCACAAGT, FAM-TTCTGACCTGAAGGCTCTGCGCG-BBQ. Reactions were carried out in 20-μL volumes containing the following: 4.4 μL QuantiTect Virus Mastermix (Qiagen, UK), 0.2 μL QuantiTect Virus RT Mix, 0.4 μM forward primer, 0.4 μM reverse primer, 0.2 μM probe, 1 μL RNA, 0.5 μL nonacetylated bovine serum albumin (BSA; 2 mg/mL; Sigma-Aldrich, UK). RT-qPCR was conducted on a QuantStudio 3 (ThermoFisher Scientific, UK) with the following cycle conditions: 50°C for 20 minutes, 95°C for 5 minutes, followed by 40 cycles of 95°C for 15 seconds and 58°C for 45 seconds.

### SARS-CoV-2 Variant Identification

Variants were analyzed by sequencing a portion of the Spike gene using the following primers: GTGTTAATCTTACAACCAGAACTCAATTAC, CACAGACTTTAATAACAACATTAGTAGCG. RT-PCR conditions were as above, except that the annealing temperature was 55°C. Sanger sequencing used the same primers (Eurofins Genomics, Germany).

### Statistical Analysis

The distribution of continuous variables was analyzed for normality using the Shapiro-Wilk test. Differences between sample types were analyzed using analysis of variance and Kruskal-Wallis test followed by Dunn’s multiple-comparison test. Spearman’s rank correlation assessed the relationship between qPCR and plaque assay results. Analyses were carried out in GraphPad Prism 6 (GraphPad Software).

## RESULTS

A total of 117 samples (44 saliva, 32 subglottic, and 41 BAL) were obtained from 25 adults admitted to the ICU at the University Hospital for Wales, a tertiary referral center. All patients had a diagnosis of SARS-CoV-2 based on nasopharyngeal swab, and none had received a SARS-CoV-2 vaccine. The median age was 59 years (range: 37 to 76 years), with a male bias (16/25, 64%). All were sedated and mechanically ventilated throughout the study, with the majority immunosuppressed as a result of treatment with dexamethasone (92%) and tocilizumab (40%) as part of their COVID-19 evidence-based therapy [10, 11].

**Figure 1.**
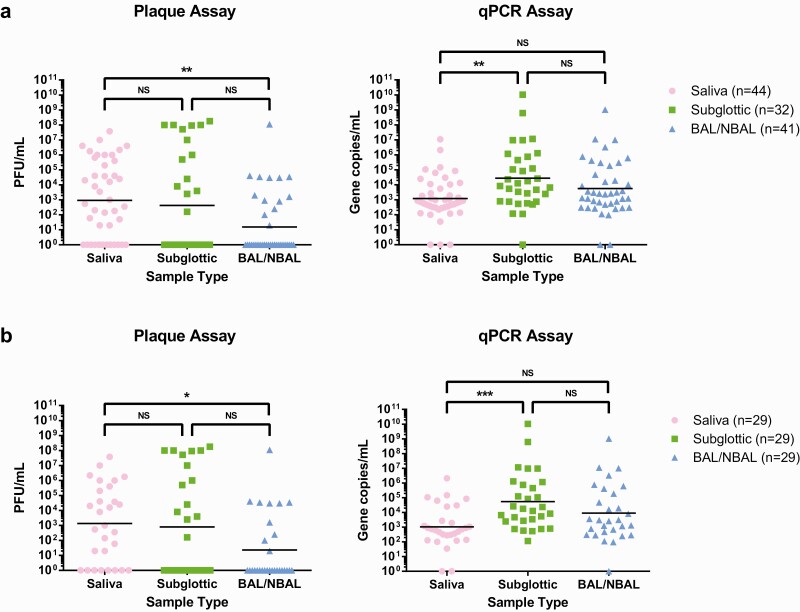
Comparisons of viable viral and gene copy load from saliva, subglottic aspirate, and bronchoalveolar lavage as determined by plaque and qPCR assays for (*a*) all samples processed in this study and (*b*) the subgroup wherein sampling time points that were incomplete (did not contain all 3 sample types) were omitted. Lines represent the geometric means. *P < .05, **P < .01, ***P < .001 (Kruskal-Wallis test with Dunn’s multiple comparison test). Abbreviations: BAL/NBAL, bronchoalveolar lavage/nondirected bronchoalveolar lavage; NS, not significant; qPCR, quantitative polymerase chain reaction.

**Figure 2.**
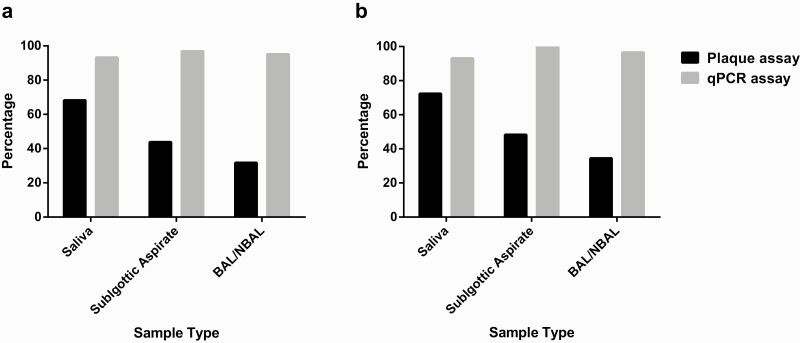
Percentage of all saliva, subglottic aspirate, and bronchoalveolar samples that were found to be positive by qPCR and plaque assays for (*a*) all samples processed in this study and (*b*) the subgroup wherein sampling timepoints that were incomplete (did not contain all 3 sample types) were omitted. Abbreviations: BAL/NBAL, bronchoalveolar lavage/nondirected bronchoalveolar lavage; qPCR, quantitative polymerase chain reaction.

To determine whether levels of virus shedding varied across sites within the respiratory tract, samples of NBAL/BAL, subglottic aspirate, and saliva were taken and assessed for RNA genome levels and live virus titers (Figure 1). It was not always possible to collect all sample types at each time point, especially where it was believed that NBAL/BAL might further compromise the patient’s respiratory capacity. To avoid bias from variable sampling times, data were analyzed across all samples (Figure 1a), and having excluded time points with incomplete data (Figure 1b). Consistent with diagnosis at admission, qPCR showed extremely high rates of positivity across all sample types (93–97%). In contrast, detection rates for live virus varied between sample types. Most saliva samples (30/44, 68%) contained live virus (Figures 1 and 2); however, this was not the case in subglottic aspirates and BAL/NBAL samples. Nevertheless, infectious virions were still detected in 14 of 32 (44%) subglottic aspirate samples and 13 of 41 (32%) BAL samples (Figures 1 and 2).

Live virus titers varied from the limit of detection (10 plaque-forming units [PFU]/mL) to extremely high (>10^8^ PFU/ml) and were significantly higher in saliva than in BAL. Across all samples, average titers reflected the chances of recovering live virus from any sample, with saliva containing the highest (1 × 10^3^ PFU/mL), while subglottic aspirates were slightly lower (2.5 × 10^2^ PFU/mL) and BAL lower still (1 × 10^1^ PFU/mL) (Figure 1). In contrast, when samples from which virus could not be isolated were excluded, subglottic aspirates contained higher titers of live virus (4.5 × 10^7^ PFU/mL) than either saliva (2.2 × 10^6^ PFU/mL) or BAL/NBAL (8.5 × 10^6^ PFU/mL). This latter result was also reflected in genome copy numbers, which were notably higher in subglottic aspirates than in saliva.

**Figure 3.**
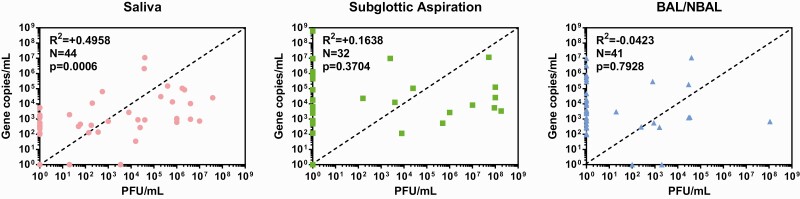
Correlation between viral load as determined by plaque assay (PFU/mL) and gene copies/mL as determined by qPCR assay. Comparisons were made between the saliva, subglottic aspirations, and BAL/NBAL sample types. The dashed line represents equal titers of the gene copy and viable viral loads (Spearman’s rank). Abbreviations: BAL/NBAL, bronchoalveolar lavage/nondirected bronchoalveolar lavage; qPCR, quantitative polymerase chain reaction.

Previous studies have demonstrated a correlation between qPCR cycle threshold (Ct) value and the chances of recovering live virus from oral swabs, with isolation of live virus becoming more infrequent as Ct values increase. In accordance with this, qPCR was clearly more sensitive than virus isolation across all sample types. However, when virus and genome titers were compared (Figure 3), we observed a moderate, significant correlation for saliva but not for subglottic aspirates or BAL/NBAL. In saliva, samples lacking live virus all had genome titers below 10^4^ copies/mL, suggesting a “cutoff” for detection of infectious virus. However, among samples containing live virus, genome titers were as low as 10^2^ copies/mL. The correlation was even weaker in BAL and subglottic aspirates, where we failed to isolate live virus from samples containing RNA levels as high as 10^9^ genomes/mL, but successfully isolated virus from samples with genome titers of 10^3^ copies/mL.

**Figure 4.**
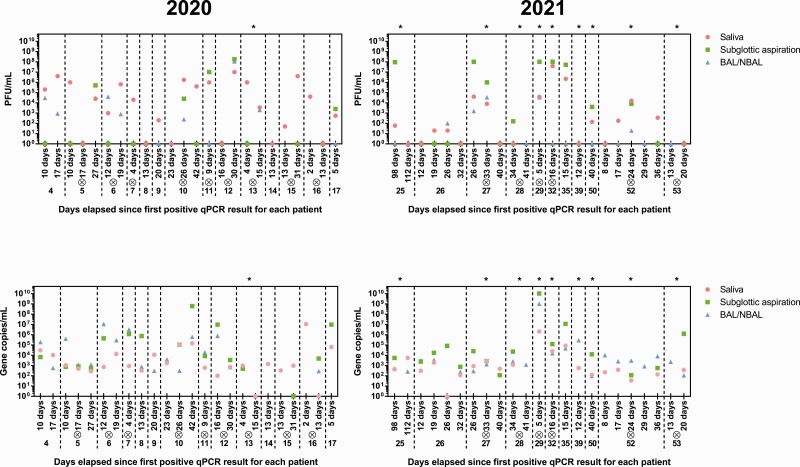
Longitudinal analyses of patient viral and gene copy loads as determined by plaque and qPCR assays, respectively. A cross inside a circle above the patient number indicates a fatal outcome for the patient. Asterisks above a patient block indicate the patient was infected with the Alpha variant (B.1.1.7). Abbreviations: BAL/NBAL, bronchoalveolar lavage/nondirected bronchoalveolar lavage; qPCR, quantitative polymerase chain reaction.

In previous data live virus was rarely detected beyond 10 days after symptom onset from oro- or nasopharyngeal swab samples, even among hospitalized patients [5]. The situation was markedly different in our cohort, where 16 of the 25 patients (64%) shed viable virus for longer than 10 days (Figure 4, Supplementary Data 1). The longest duration of shedding was 98 days, while the majority of patients (14/25, 56%) shed virus for 20 days or longer. Saliva and subglottic aspirate tended to remain positive for longer than BAL, in accordance with our previous observation that BAL was the sample least likely to contain viable virus.

While this study was underway, the Alpha variant of concern began to spread. We therefore sequenced the Spike gene to determine whether the isolation of viable virus differed based on strain. No clear differences were seen in the longevity of virus isolation. Furthermore, no correlation was observed between viral load and patient outcome.

When viable viral and gene copy loads from each patient were compared longitudinally, the highest viral titers across all patients were recorded from subglottic aspiration samples. However, overall, saliva provided a better indication of infection; whenever live virus was isolated from any sample at any time point, saliva at that time point always contained live virus. In contrast, by qPCR, sample type was largely irrelevant for determining positivity. There were, however, differences in viral load by qPCR, with subglottic aspirates often containing higher titers than saliva.

## DISCUSSION

Current National Health Service guidance states that isolation precautions can be discontinued in individuals with SARS-CoV-2 infection 10 days after symptom onset [15], while the CDC recommends extending this for up to 20 days after symptom onset in those with severe illness [12]. Our study clearly demonstrates that ICU patients frequently excrete high titers of infectious SARS-CoV-2 for periods far exceeding these recommendations. Viral titers from saliva, subglottic aspirate, and BAL/NBAL can reach more than 10^7^ PFU/mL in some patients. This was not variant specific as individual patients infected by either Alpha, or earlier variants, shed these high titers of infective virus. Our study also highlights the inadequacy of qPCR in determining the point during the infection course when an intubated patient ceases to present an infection risk to hospital staff; this was particularly true for airway and lower respiratory tract samples, where PCR positivity was poor at predicting live virus. Furthermore, in contrast to studies using oral swabs in hospitalized patients [16], Ct values of ICU patients were not a good predictor of the presence of live virus, a problem that has been highlighted in previous studies investigating discrepancies between RT-PCR and symptomatic infection [17–19].

This adds to an increasing body of evidence relating to the extended duration of live virus shedding from ventilated patients with COVID-19. van Kampen et al [20] grouped patients from wards and the ICU and showed that live virus can be isolated from the sputum and upper respiratory tract samples of patients with severe or critical COVID-19 for more than 10 days; however, this was only in a small proportion (10%) of samples, with the median duration of shedding being 8 days from symptom onset. Furthermore, whether the samples containing live virus were sputum or upper respiratory samples was not defined, whether long-term shedding correlated with mechanical ventilation was not examined, and only 1 patient excreted live virus for up to 20 days. A second study examined patients in the ICU, of whom 72% were mechanically ventilated [21]. They found that patients excreted virus for a median of 13 days from the upper respiratory tract, and only 2 remained positive up to 20 days. We observed much longer durations of shedding, with most patients excreting virus for more than 20 days, up to a maximum of 98 days. We also isolated live virus from a much greater proportion of patients (87%) compared with previous studies (17.8% and 7%, respectively). This may reflect the clinical characteristics of our cohort; all were mechanically ventilated and the majority had failed steroid therapy on the wards (whereas earlier cohorts were steroid naive), which is reflected in their high mortality (64%). It may also reflect our use of saliva (sample type was not specified in previous studies [20, 21]); however, nasopharyngeal swabs are common. These capture a small sample quantity and dilute it further in transport medium. In addition, previous studies did not isolate virus on cells expressing human ACE2 and TMPRSS2; expression of these proteins represents a more biologically relevant target cell and significantly enhances virus detection [13]. This highlights the need to use sensitive methodologies and repeat sampling before concluding that patients are not secreting live virus. Our data also extend these previous studies by correlating upper and lower respiratory tract samples and demonstrating that live virus shedding also occurs from the lower respiratory tract for extended durations.

Only 1 other study has titrated live virus from clinical samples. Differences in sample and cell type can alter viral titer readouts; nevertheless, this study demonstrated titers of 5 × 10^6^ and 4 × 10^6^ PFU/mL in nasopharyngeal swabs from 2 patients [22]. Thus, despite their prolonged shedding, titers in ICU patients may not be substantially different from those with milder disease. Mouth swabs are commonly used to diagnose SARS-CoV-2 infection and may be interpreted as a surrogate for shedding of live virus. However, the respiratory droplets that transmit virus have been assumed to arise from both the upper and lower respiratory tract. Previous studies have compared genome loads in BAL compared with mouth swabs using qPCR [23, 24]. In agreement with these studies, we find that PCR is largely concordant between upper and lower respiratory tract samples; thus, BAL/NBAL do not offer advantages over the more practical upper respiratory samples for diagnosis. However, in contrast to viral genome, live virus was much more common in saliva than BAL/NBAL, suggesting that the upper respiratory tract is more likely to be a source of infectious virus than the lower respiratory tract. This is consistent with previous reports that showed there is independent replication of virus in the upper and lower airways [25]. However, it may also reflect the chances of virus being inactivated in a sample containing high levels of mucus and other proteolytic enzymes, and the volume of fluid used to lavage the lungs’ parenchyma. Titers in BAL/NBAL may therefore underestimate the true situation. Nevertheless, it is clear that cell-free live virus is capable of reaching extremely high titers in the lungs, and is a potential source of transmissible virus in a proportion of patients. This discordance between titers of infectious virus and viral genomes in BAL/NBAL highlights the advantage of measuring infectious viral load directly by plaque assay. Commonly implemented indirect viral load measurement methods, such as measuring gene copies and subsequently confirming the sample to contain infectious virions by observing cytopathic effects (CPE) on cultured cells [20, 21, 25–27], would have resulted in over- and underestimation of infectious viral load in numerous samples.

In a proportion of samples, genome titers were lower than live virus titers. This likely reflects the difficulty of extracting RNA from a highly proteolytic sample, and the need to process the sample to exclude carry-through inhibitors—problems that are reduced in nasopharyngeal swabs that most studies use. All qPCR reactions were controlled by RNase P to ensure that inhibitors did not affect results, and this is reflected in the fact that nearly all samples were positive for viral RNA. Nevertheless, the higher processing requirements, and the lability of RNA, may result in the genome copy number being an underrepresentation of the in vivo situation. Despite this, the genome load in our cohort was similar to those previously reported from oropharyngeal [16] and saliva [28] samples in hospitalized patients.

Our study demonstrates that qPCR is not a robust indicator of viable viral shedding in critically ill patients, irrespective of sample type. Patients in the ICU infected with SARS-CoV-2 tend to be prolonged shedders, excreting virus for far beyond the time periods specified in current guidelines, and virus titers can be extremely high in both the upper and lower respiratory tracts. This may be a consequence of infection-induced immunosuppression and/or the use of steroids and/or interleukin 6 (IL-6) blockade to limit tissue damage. This information is important for decision making around cohorting patients, de-escalation of personal protective equipment, and undertaking potential aerosol-generating procedures, particularly given the threat of new variants such as Omicron that have higher transmission rates and greater vaccine escape potential. It also supports the continued use of oral antiseptics in these patients; antiseptics such as chlorhexidine are used routinely to reduce ventilator-associated pneumonia [29]. These may also have a role to play in minimizing nosocomial transmission, although formulations containing surfactants are likely to be most effective [30]. Larger multicenter cohorts are now needed to determine the clinical features that correlate with long-term shedding (eg, age, humoral and cellular immune responses, specific treatments), and to assess whether the use of monoclonal antibody therapies, vaccination, and antivirals can reduce persistent shedding. Our study also highlights the need for more robust, practical assays for the determination of viable viral shedding in healthcare settings.

## Supplementary Data

Supplementary materials are available at *Clinical Infectious Diseases* online. Consisting of data provided by the authors to benefit the reader, the posted materials are not copyedited and are the sole responsibility of the authors, so questions or comments should be addressed to the corresponding author.

ciac170_suppl_Supplementary_Data

ciac170_suppl_Supplementary_Legends

## Notes

***Acknowledgments.*** The authors thank John Watkins (Cardiff University) for statistical advice.

***Financial support*.** This work was supported by funding from the Medical Research Council (grant numbers MR/V028448/1, MR/S00971X/1), Wellcome Trust (grant number 204870/Z/16/Z), Welsh Government Ser Cymru Scheme, Welsh Clinical Academic Training (WCAT) Fellowship Scheme, and Accelerate Wales. R. S. reports funding from Biotechnology and Biological Sciences Research Council (BBSRC).

***Potential conflicts of interest.*** M. P. W. reports being a co-applicant on a grant application to his institution from UK Research and Innovation-Biotechnology and Biological Sciences Research Council (UKRI-BBSRC) Covid-19 Agile Response Call—“Mapping the lipid envelope composition of SARS-CoV-2 for reducing transmission, thrombosis, and inflammation”—co-applicant £597,540 and receiving payment for lectures from Gilead and Fisher & Paykel. M. P. reports payments to his institution from the Association of Clinical Pathologists (ACP) UK Career Development Award outside of the submitted work. All other authors report no potential conflicts.

## References

[1] World Health Organization. Coronavirus disease (COVID-19) situation reports. Available at: https://www.who.int/emergencies/diseases/novel-coronavirus-2019/situationreports. Accessed 15 June 2021.

[2] Wu Z , McGoogan JM. Characteristics of and important lessons from the coronavirus disease 2019 (COVID-19) outbreak in China. JAMA 2020; 323:1239–42. 32091533 10.1001/jama.2020.2648

[3] Guan WJ , Ni ZY, Hu Y, et al Clinical characteristics of coronavirus disease 2019 in China. N Engl J Med 2020; 382:1708–20. 32109013 10.1056/NEJMoa2002032 PMC7092819

[4] Bak A , Mugglestone MA, Ratnaraja NV, et al SARS-CoV-2 routes of transmission and recommendations for preventing acquisition: joint British Infection Association (BIA)Healthcare Infection Society (HIS), Infection Prevention Society (IPS) and Royal College of Pathologists (RCPath) guidance. J Hosp Infect 2021; 114:79–103. 33940093 10.1016/j.jhin.2021.04.027 PMC8087584

[5] Cevik M , Tate M, Lloyd O, Maraolo AE, Schafers J, Ho A. SARS-CoV-2, SARS-CoV, and MERS-CoV viral load dynamics, duration of viral shedding, and infectiousness: a systematic review and meta-analysis. Lancet Microbe 2021; 2:e13–22. 33521734 10.1016/S2666-5247(20)30172-5 PMC7837230

[6] Horby P , Lim WS, Emberson JR, et al; RECOVERY Collaborative Group. Dexamethasone in hospitalized patients with Covid-19. N Engl J Med 2021; 384:693–704. 32678530 10.1056/NEJMoa2021436 PMC7383595

[7] Benotmane I , Gautier- Vargas G, Wendling M-J, et al In-depth virological assessment of kidney transplant recipients with COVID- 19. Am J Transplant 2020; 20:3162–72. 32777130 10.1111/ajt.16251 PMC7436721

[8] Benotmane I , Risch S, Doderer-Lang C, Caillard S, Fafi-Kremer S. Long-term shedding of viable SARS-CoV-2 in kidney transplant recipients with COVID-19. Am J Transplant 2021; 21:2871–5. 33961334 10.1111/ajt.16636 PMC8222938

[9] Kemp SA , Collier DA, Datir RP, et al; CITIID-NIHR BioResource COVID-19 Collaboration; COVID-19 Genomics UK (COG-UK) Consortium. SARS-CoV-2 evolution during treatment of chronic infection. Nature 2021; 592:277–82. 33545711 10.1038/s41586-021-03291-y PMC7610568

[10] Ponsford MJ , Shillitoe BMJ, Humphreys IR, Gennery AR, Jolles S. COVID-19 and X-linked agammaglobulinemia (XLA)—insights from a monogenic antibody deficiency. Curr Opin Allergy Clin Immunol 2021; 21:525–34. 34596095 10.1097/ACI.0000000000000792

[11] Remy KE , Mazer M, Striker DA, et al Severe immunosuppression and not a cytokine storm characterizes COVID-19 infections. JCI Insight 2020; 5:e140329. 10.1172/jci.insight.140329 PMC7526441 32687484

[12] Centers for Disease Control and Prevention. Interim guidance on ending isolation and precautions for adults with COVID-19. March 16, 2021. Available at: https://www.cdc.gov/coronavirus/2019-ncov/hcp/duration-isolation.html?CDC_AA_refVal=https%3A%2F%2Fwww.cdc.gov%2Fcoronavirus%2F2019-ncov%2Fcommunity%2Fstrategy-discontinue-isolation.html. Accessed 18 November 2021.

[13] Rihn SJ , Merits A, Bakshi S, et al A plasmid DNA-launched SARS-CoV-2 reverse genetics system and coronavirus toolkit for COVID-19 research. PLoS Biol 2021; 19:e3001091. 33630831 10.1371/journal.pbio.3001091 PMC7906417

[14] US Food and Drug Administration. LabCorp COVID-19 RT-PCR Test EUA summary, accelerated emergency use authorization (EUA) summary COVID-19 RT-PCR TEST. Silver Spring, MD: US Food and Drug Administration, 2020.

[15] National Health Service . How long to self-isolate. November 11, 2021. Available at: https://www.nhs.uk/conditions/coronavirus-covid-19/self-isolation-and-treatment/how-long-to-self-isolate/. Accessed 18 November 2021.

[16] Fajnzylber J , Regan J, Coxen K, et al SARS-CoV-2 viral load is associated with increased disease severity and mortality. Nat Commun 2020; 11:5493. 33127906 10.1038/s41467-020-19057-5 PMC7603483

[17] Winichakoon P , Chaiwarith R, Liwsrisakun C, et al Negative nasopharyngeal and oropharyngeal swabs do not rule out COVID-19. J Clin Microbiol 2020; 58:e00297–20. 32102856 10.1128/JCM.00297-20 PMC7180262

[18] Wang Y , Kang H, Liu X, et al Combination of RT-qPCR testing and clinical features for diagnosis of COVID-19 facilitates management of SARS-CoV-2 outbreak. J Med Virol 2020; 92:538–9. 32096564 10.1002/jmv.25721 PMC7233289

[19] Tahamtan A , Ardebili A. Real-time RT-PCR in COVID-19 detection: issues affecting the results. Expert Rev Mol Diagn 2020; 20:453–4. 32297805 10.1080/14737159.2020.1757437 PMC7189409

[20] van Kampen, JJA., van de Vijver, DAMC., Fraaij, PLA. et al Duration and key determinants of infectious virus shedding in hospitalized patients with coronavirus disease-2019 (COVID-19). Nat Commun 2021; 12:267. 33431879 10.1038/s41467-020-20568-4 PMC7801729

[21] Funk DJ , Bullard J, Lother S, et al Persistence of live virus in critically ill patients infected with SARS-COV-2: a prospective observational study. Crit Care 2022; 26:10. 34983614 10.1186/s13054-021-03884-z PMC8724747

[22] Shrivastava S , Patil HP, Mhaske ST, et al Isolation and genetic characterization of SARS-CoV-2 from Indian patients in a single family without H/O travel abroad. Virus Genes 2021; 57:245–9. 33683658 10.1007/s11262-021-01826-z PMC7938031

[23] Abid MB , Chhabra S, Buchan B, et al Bronchoalveolar lavage-based COVID-19 testing in patients with cancer. Hematol Oncol Stem Cell Ther 2021; 14:65–70. 33058787 10.1016/j.hemonc.2020.09.002 PMC7543702

[24] Gao CA , Cuttica MJ, Malsin ES, Argento AC, Wunderink RG, Smith SB. NU COVID investigators. comparing nasopharyngeal and BAL SARS-CoV-2 assays in respiratory failure. Am J Respir Crit Care Med 2021; 203:127–9. 33125256 10.1164/rccm.202008-3137LE PMC7781122

[25] Wölfel R , Corman VM, Guggemos W, et al Virological assessment of hospitalized patients with COVID-2019. Nature 2020; 581:465–9. 32235945 10.1038/s41586-020-2196-x

[26] Kim MC , Cui C, Shin KR, et al Duration of culturable SARS-CoV-2 in hospitalized patients with Covid-19. N Engl J Med 2021; 384:671–3. 33503337 10.1056/NEJMc2027040 PMC7934323

[27] Li Q , Zheng XS, Shen XR, et al Prolonged shedding of severe acute respiratory syndrome coronavirus 2 in patients with COVID-19. Emerg Microbe Infect 2020; 9:2571–7. 10.1080/22221751.2020.1852058 PMC7734137 33196399

[28] To KK , Tsang OT, Leung WS, et al Temporal profiles of viral load in posterior oropharyngeal saliva samples and serum antibody responses during infection by SARS-CoV-2: an observational cohort study. Lancet Infect Dis 2020; 20:565–74. 32213337 10.1016/S1473-3099(20)30196-1 PMC7158907

[29] Wittekamp BH , Plantinga NL. Less daily oral hygiene is more in the ICU: not sure. Intensive Care Med 2021; 47:331334–333. 10.1007/s00134-021-06359-5 PMC7870028 33558968

[30] Saud Z , Tyrrell VJ, Zaragkoulias A, et al The SARS-CoV2 envelope differs from host cells, exposes pro-coagulant lipids, and is disrupted in vivo by oral rinses. J Lipid Res 2022; doi:10.1016/j.jlr.2022.100208 PMC9010312 35436499

