# Supplementary material for: Mechanically Ventilated Patients Shed High-Titer Live Severe Acute Respiratory Syndrome Coronavirus 2 (SARS-CoV-2) for Extended Periods From Both the Upper and Lower Respiratory Tract
Source: Clin Infect Dis. 2022 Mar 1;75(1):e82–8. doi: 10.1093/cid/ciac170 (PMC9129116; doi:10.1093/cid/ciac170)
Supplement: ciac170_suppl_Supplementary_Legends [file ciac170_suppl_supplementary_legends.docx]

**Supplementary data
1a.** Individual patient baseline characteristics and treatment regimen.

**1b.** Individual sample viral load, gene copy load, variant sequence, RNAseP Ct, and days exhibiting positive assay result.

**1c.** Individual sample qPCR Ct values and gene copy load calculations.
